# Research on the Correlation Between the Chemical Components and the Macroscopic Properties of Asphalt Binder

**DOI:** 10.3390/ma18030610

**Published:** 2025-01-29

**Authors:** Zhihao Li, Xuejuan Cao, Jue Li, Xiaoyu Yang

**Affiliations:** 1 School of Civil Engineering, Chongqing Jiaotong University, Chongqing 400074, China; 611210111003@mails.cqjtu.edu.cn; 2 School of Material Science and Engineering, Chongqing Jiaotong University, Chongqing 400074, China; caoxj@cqjtu.edu.cn; 3 College of Traffic & Transportation, Chongqing Jiaotong University, Chongqing 400074, China; yxyss868@mails.cqjtu.edu.cn

**Keywords:** road engineering, SARA fractions, asphalt binder, correlation analysis

## Abstract

The chemical composition of asphalt binder is closely related to its macroscopic properties, and as an important road building material, its performance directly affects the service performance of asphalt binder pavement. Saturate, aromatic, resin, and asphaltene are the four most common chemical components of asphalt binders, collectively known as the SARA components. The SARA components are used to establish the corresponding relationship between the chemical composition and the macroscopic properties of asphalt binder, which is of great significance for further research on and development of high-performance asphalt pavement materials. This study used eight types of virgin asphalt binders as raw materials, labeled A–H. Firstly, the thin-layer chromatography–flame ionization detection (TLC-FID) method was used to test the SARA contents of the different asphalt binders. Then, the conventional, rheological, and low-temperature properties of the different binders were tested. Finally, gray relational analysis (GRA) and Pearson correlation analysis (PCA) were used to study the correlation between the asphalt binder’s SARA content and its macroscopic properties. The results indicate that the contents of asphaltenes and resins are crucial in determining the high-temperature performance of asphalt binder. By adjusting the ratio of these components, the high-temperature performance of asphalt binder can be optimized. An increase in the content of heavy components, particularly asphaltenes, negatively affects the low-temperature performance of asphalt binder. In contrast, a higher aromatic content enhances its low-temperature performance.

## 1. Introduction

In recent decades, rapid economic development, the expansion of construction projects, and increased traffic volumes have led to a sharp rise in the number of heavy-load vehicles. Consequently, the demand for improved road performance has increased significantly [1]. As a widely used material for high-performance pavements, asphalt binder has a complex chemical composition [2], primarily consisting of carbon, hydrogen, and heteroatoms such as nitrogen, sulfur, and oxygen. Additionally, trace metals, including vanadium and nickel, have also been detected [3,4,5,6]. Moreover, a “phase equilibrium” typically exists within the asphalt binder structure, where polar and non-polar substances, homogeneous and heterogeneous components, and small and large molecular compounds are balanced [7]. The delicate balance of components in asphalt binder plays a crucial role in its macroscopic properties. An optimal compositional structure ensures stable mechanical performance and extends its service life [8]. Therefore, exploring and establishing the relationship between the microscopic composition of asphalt binder and its macroscopic properties, with the aim of enhancing its road performance, has become a central focus of the ongoing development of the road industry.

Asphalt binder is a mixture of complex hydrocarbons of varying molecular weights, along with their non-metallic derivatives. It is a high-viscosity organic binder, obtained either naturally or as a final product during petroleum refining [9]. Asphalt binder typically appears as a black or brown solid or liquid. Given the diversity and complexity of asphalt binder’s molecular structure, current technological methods face significant challenges in accurately determining its precise structure and directly correlating it with engineering properties. To facilitate research, the microscopic composition of asphalt binder is commonly categorized according to chemical structural similarities. In the early 20th century, Richardson et al. [10] classified the components of asphalt binder into asphaltenes and maltenes, with asphaltenes identified as the most polar component of the asphalt binder structure. In later studies, researchers further separated maltenes into oil and resin fractions using extraction methods, a process commonly referred to as the three-component separation of asphalt binder [11]. The standard method for separating the chemical components of asphalt binder, widely used today, was proposed by Corbett et al. [12]. This method employs column chromatography to further separate maltenes into saturates, aromatics, and resins. The components of asphalt binder, commonly known as the SARA fractions, include saturates, aromatics, resins, and asphaltenes. However, the Corbett separation method has two drawbacks in terms of the actual testing process: (i) the process is complex and time-consuming; and (ii) it is prone to human error and demands a high level of operator expertise. To overcome these challenges, researchers developed thin-layer chromatography–flame ionization detection (TLC-FID), which provides a technological foundation for the rapid separation and proportional detection of SARA fractions [13].

Both domestic and international researchers have extensively studied the correlation between the chemical composition, represented by the SARA fractions, and the performance of asphalt binders [14,15]. Japanese researchers conducted a preliminary quantification of the relationship between the softening point of asphalt binder and the proportions of its four components, using SARA fraction separation experiments and linear regression analysis. Mansourkhaki et al. [16,17] performed SARA fraction separation and rheological tests on various recycled asphalt binders. They identified asphaltenes as a critical factor in determining asphalt binder’s high-temperature rutting resistance. Furthermore, they concluded that the asphaltene content is a more reliable indicator for preliminarily assessing high-temperature performance than the colloidal stability index or asphaltene index. Tian et al. [18] investigated the relationship between the SARA fractions of asphalt binder and the critical cracking temperature (T_CR_) using a bending beam rheometer (BBR). Their findings indicated a strong correlation between the content of asphaltenes and aromatics and the T_CR_, with asphaltenes showing a positive correlation, and aromatics, a negative one. In contrast, the correlation between saturates and low-temperature performance was relatively weak.

The SARA components are widely recognized as key indicators for analyzing the relationship between the chemical composition of asphalt binder and its macroscopic performance [19,20]. Global researchers have conducted extensive studies on the correlation between the chemical composition of asphalt binder, represented by the SARA components, and its macroscopic properties, including engineering performance indicators and rheological characteristics [21,22]. Unlike traditional qualitative or non-directional studies, this research not only examines the correlation between variables but also explores the influence of the chemical composition on the macroscopic performance. Simultaneously, the most significant factors are identified using both GRA and PCA, thereby clarifying the chemical composition characteristics of asphalt binder materials tailored to specific requirements. This information can then be used to select, adjust, and optimize asphalt binder material designs, thereby laying the foundation for the development of high-performance pavement materials. This study used eight types of virgin asphalt binders as raw materials. First, the content of the SARA fractions was determined using TLC-FID. Then, a series of laboratory tests were conducted to evaluate the macro-properties of the different asphalt binders. Finally, the correlations between the chemical components of the asphalt binder and its macro-properties were analyzed using GRA and PCA. The findings provide a theoretical foundation for the further development of high-performance asphalt pavement materials.

## 2. Materials and Methods

### 2.1. Virgin Asphalt Binders and Instruments

The test numbers and basic information on the eight types of virgin asphalt binders are shown in Table 1, and the test reagents are presented in Table 2.

### 2.2. Determination of the SARA Fractions of Asphalt Binder

**Figure 1:**
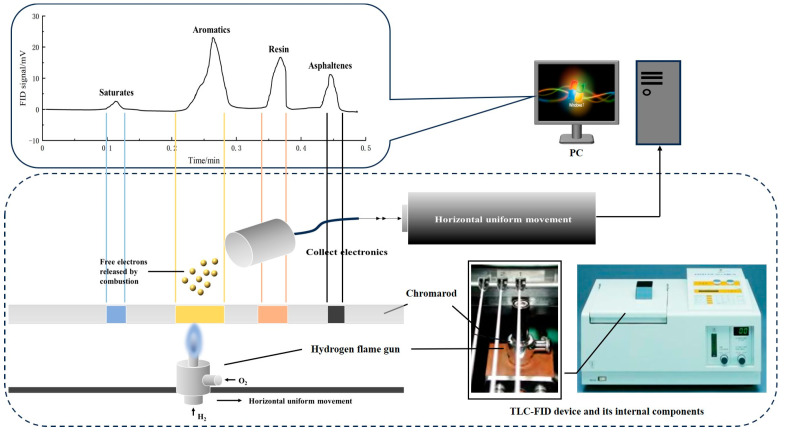
Test principle of the TLC-FID method.

The current solvent precipitation and column chromatography method (also known as the Corbett method) for separating the SARA fractions is characterized by slow separation, being a complex process, high contamination, significant susceptibility to human error, and poor reproducibility of the results [23]. This study employed thin-layer chromatography–flame ionization detection (TLC-FID) to analyze the components of various asphalt binders. The fundamental principles of the test are outlined in Figure 1. The method relies on the principle that the SARA fractions of asphalt binder diffuse to varying extents in a specific solvent, resulting in distinct chromatographic positions for each component. The asphalt binder was efficiently separated into its SARA fractions on a dedicated chromatographic rod, which was then inserted into a TLC-FID analyzer (model MK-6020, Iatroscan, Tokyo, Japan) for further testing. The mechanical drive system moved the hydrogen flame torch at a constant horizontal speed, ensuring that the entire chromatographic rod passed uniformly through the center of the hydrogen flame. This allowed each component to combust and crack, releasing free electrons. The electrodes collected the free electrons, converting them into a current signal proportional to the content of each component. This produced a voltage signal curve that changed in response to the movement of the hydrogen flame, as illustrated in the upper-left section of Figure 1. The content of each component was determined by calculating the ratio of the area under each voltage peak to the total area.

The preparation of the chromatographic rod for the asphalt binder SARA fractions involved four stages [24,25]. Phase 1: The asphalt binder was dissolved in a 30 mg/mL toluene solution, and 0.8 μL to 1.2 μL of the resulting asphalt binder–toluene solution was then applied to the chromatographic rod. Phase 2: The chromatographic rod was vertically placed into an unfolding platform containing heptane solution, allowing the asphalt binder–toluene mixture to fully diffuse within the heptane environment. Phase 3: The chromatographic rod, containing the solution, was placed in an oven (model DHG-9023A, Wuxi Maret, Wuxi, China) at 80 °C for drying to stabilize the chromatographic material. Subsequently, the rod was transferred to a toluene solution diffuse platform for a second diffuse, followed by immediate drying. Phase 4: The dried chromatographic rod was placed in a toluene–anhydrous ethanol (55:45) solution diffuse platform for the third diffuse, followed by immediate drying. Once dried, the preparation of the chromatographic rod was complete. The prepared chromatographic rod was then inserted into the TLC-FID analyzer to determine the content of the asphalt binder’s SARA fractions.

### 2.3. Conventional Properties Test

According to JTG E20 T0604 [26], the penetration of the asphalt binder samples was tested using a penetration tester (model A5010, Delite Technology Co., Ltd., Shenzhen, China) at a temperature of 25 °C, with a standard needle mass of 100 g and a vertical penetration time of 5 s. The test results were accurate to 0.1 mm, and parallel tests were conducted at least 3 times. The distance between the test points and the edge of the sample dish was not less than 10 mm. According to JTG E20 T0605 [26], the ductility of the asphalt binder samples was tested using a ductility tester (model SY-1.5B, Weichuang Experimental Instrument Co., Ltd., Henan, China) at a temperature of 10 °C and a tensile speed of 5 cm/min. Parallel tests were conducted at least three times. According to JTG E20 T0606 [26], the softening point of the asphalt binder samples was tested with a softening point tester (model SYD-2806F, Shengxing Instrument Equipment Co., Ltd., Shanghai, China) using the global method. An asphalt binder sample was placed in a copper ring of a specific size (diameter, 16 mm; height, 6 mm), and a standard steel ball was placed on top of the sample. The sample was then heated at a specified rate (5 °C/min) until the asphalt binder softened. Parallel tests were conducted at least twice.

### 2.4. Dynamic Shear Rheometer Test

A dynamic shear rheometer (model Smart Pave 102, Anton Paar GmbH, Graz, Austria) in temperature sweep mode was employed to measure the complex shear modulus (G*) and phase angle (δ) of the asphalt binder samples. Additionally, multiple stress creep recovery (MSCR) tests were performed to determine the irreversible creep compliance (J_nr_) of the asphalt binder [27]. Temperature sweep mode: The temperature range was from 52 °C to 82 °C, with a strain control of 12% and a frequency of 10 rad/s. A 25 mm parallel plate was used, with the plate gap set to 1 mm. MSCR: The test was conducted at a temperature of 64 °C, with stress levels of 0.1 kPa and 3.2 kPa. The loading creep lasted for 1 s, followed by a 9 s unloading recovery, with a total of 10 cycles for each stress level. A 25 mm parallel plate was used, with the plate gap set at 1 mm.

### 2.5. Bending Beam Rheometer Test

The creep stiffness (S) and creep rate (m) of the various asphalt binder samples were evaluated using a bending beam rheometer (model BBR, Shanghai Shenrui, Shanghai, China). The experiment used a double-sided support middle loading mode to load the asphalt binder beams, with the dimensions of 127 ± 2 mm in length, 6.35 ± 0.05 mm in thickness, and 12.70 ± 0.05 mm in width. The distance between the support points on both sides was 102 mm. The experimental temperature was −12 °C, the load was 980 ± 50 mN, and the load application time was 240 s. The S and m values were measured and analyzed at 60 s.

## 3. Results

### 3.1. SARA Fractions of Asphalt Binder

**Figure 2:**
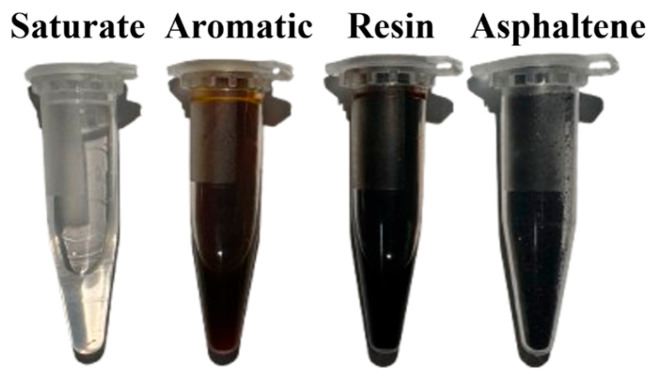
SARA fractions of asphalt binder.

At room temperature, saturate generally appears as a colorless, transparent, viscous oil, as shown in Figure 2. It is mainly a mixture of branched alkanes and cycloalkanes, with very little impure content and almost zero aromaticity. It softens the micelles formed by resin and asphaltene through synergistic action with the aromatic fractions [12,28]. Due to its chemical inertness, it maintains a relatively stable state during aging, and its content in binders is usually 2–25%. Aromatic is the main component of asphaltene dispersion media, appearing yellow or brown at room temperature, as shown in Figure 2. Aromatic fractions are mainly composed of non-polar carbon chains, which is the main reason for their strong activity and easy oxidation [29]. Usually, their content in binders is 20–50%. Resin, also known as polar aromatic hydrocarbons, is a strongly polar component with poor chemical stability and antioxidant capacity. It generally appears in a dark brown solid or semi-solid state at room temperature, as shown in Figure 2. It is mainly composed of carbon, hydrogen, and other atoms, with a few heteroatoms [30]. Research has shown that the role of resin is similar to that of a dispersant for asphaltene, which has a significant impact on the adhesion and plasticity of asphalt binder [31,32]. Its content in binder is usually 25–60%, and the ratio of asphaltene to resin greatly controls the formation of the asphalt binder’s colloid structure, playing an important role in the entire colloid system. Asphaltene is generally defined as a black amorphous solid that is insoluble in non-polar hydrocarbon solvents such as n-hexane. As shown in Figure 2, asphaltene contains a large number of heteroatoms (nitrogen, sulfur, nickel, etc.) and is often regarded as a complex aromatic substance with high polarity and high molecular weight [33,34]. As the core of the colloidal structure, asphaltene undergoes binding effects at high concentrations, so its content has a significant impact on the rheological properties of asphalt binder. Typically, its content in binder is 5–30%.

**Figure 3:**
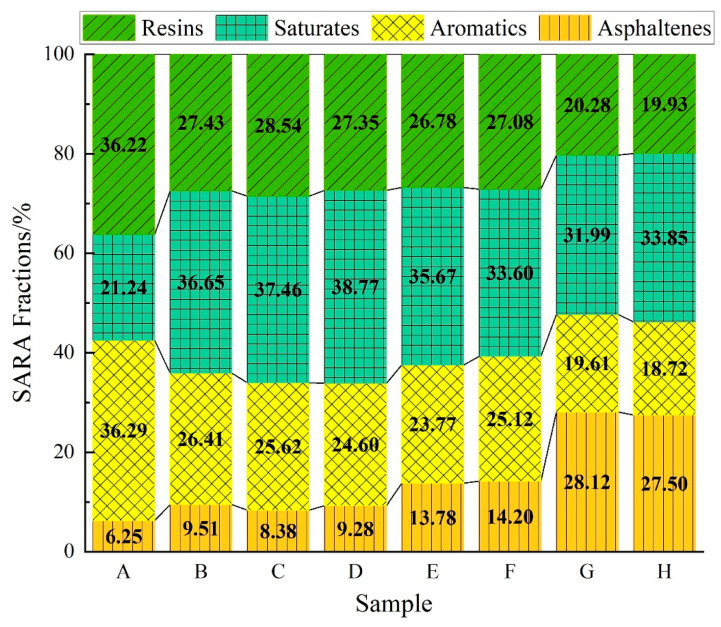
SARA fraction content.

This study employed TLC-FID to analyze the SARA fractions of eight different virgin asphalt binders, with the results presented in Table 3 and Figure 3. As shown in Figure 3, the content of the SARA fractions varies considerably across the asphalt binder types, primarily due to differences in the asphalt binder composition and processing methods. The experimental data reveal the top three resin contents among the eight virgin asphalt binders, ranked as follows: Sample A (36.22%) > Sample C (28.54%) > Sample B (27.43%). The top three saturate contents were ranked in the following order: Sample D (38.77%) > Sample C (37.46%) > Sample B (36.65%). The top three aromatic contents were ranked in the following order: Sample A (36.29%) > Sample B (26.41%) > Sample C (25.62%). The top three asphaltene contents were ranked in the following order: Sample G (28.12%) > Sample H (27.5%) > Sample F (14.2%).

### 3.2. Conventional Properties of Asphalt Binder

**Figure 4:**
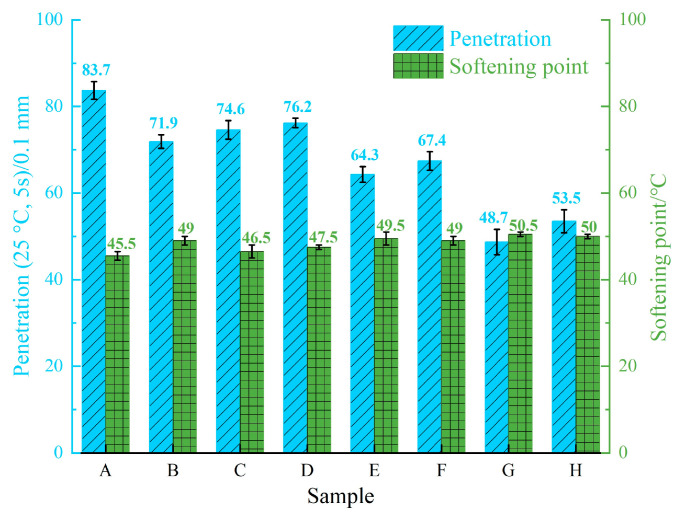
Test results of the asphalt binder penetration and softening point.

**Figure 5:**
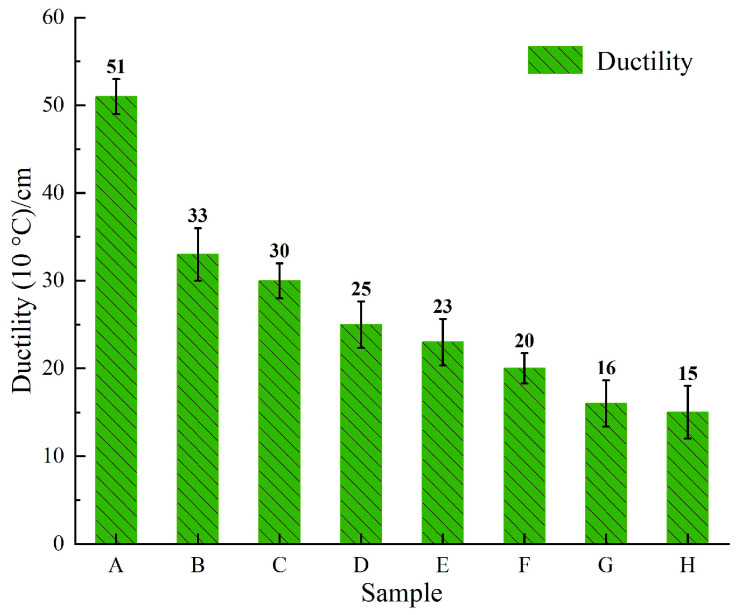
Test results of the asphalt binder ductility.

In accordance with the “Standard Test Methods of Bitumen and Bituminous Mixtures for Highway Engineering” (JTG E20-2011) [26], the conventional property tests were performed on the eight virgin asphalt binders, and the results are presented in Figure 4 and Figure 5. As shown in Figure 4 and Figure 5, Sample A exhibits the conventional properties for the 90^#^ asphalt binder specification (penetration/0.1 mm: 80–100; softening point/°C: 42–52; ductility/cm: ≥45), Samples B to F meet the specifications for 70^#^ asphalt binder (penetration/0.1 mm: 60–80; softening point/°C: 44–57; ductility/cm: ≥20), and Samples G and H conform to the standards for 50^#^ asphalt binder across conventional properties (penetration/0.1 mm: 40–860; softening point/°C: 45–58; ductility/cm: ≥15). The analysis of the charts reveals that both penetration and ductility are positively correlated with the resin content, negatively correlated with the saturate content, positively correlated with the aromatic content, and negatively correlated with the asphaltene content. The correlation trend of the softening point with the SARA fraction content is the inverse of that observed for penetration and ductility.

### 3.3. High-Temperature Performance of Asphalt Binder

**Figure 6:**
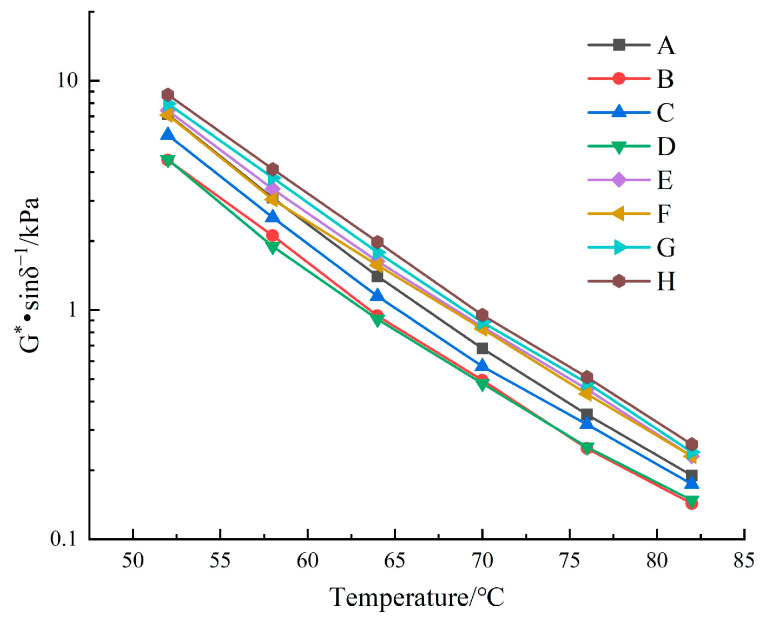
Temperature variation curve of the rutting factor.

The complex shear modulus (G*) and phase angle (δ) of the various asphalt binder samples were measured using a DSR in temperature sweep mode. The rutting factor (G*/sinδ) was subsequently calculated to assess the deformation resistance of the asphalt binder materials at elevated temperatures [35,36]. The experimental results are presented in Table 4, and the temperature variation curve of the rutting factor is shown in Figure 6.

As shown in Table 4, the value of G* decreases significantly with an increasing temperature for all the types of asphalt binder. This demonstrates that the stability of asphalt binder materials is highly sensitive to temperature fluctuations. The value of δ shows the opposite trend, suggesting that as the temperature of the asphalt binder increases, it gradually shifts from exhibiting elastic to viscous behavior. As shown in Figure 6, at a constant temperature, the rutting factor rankings are as follows: Sample H > Sample G > Sample E.

**Figure 7:**
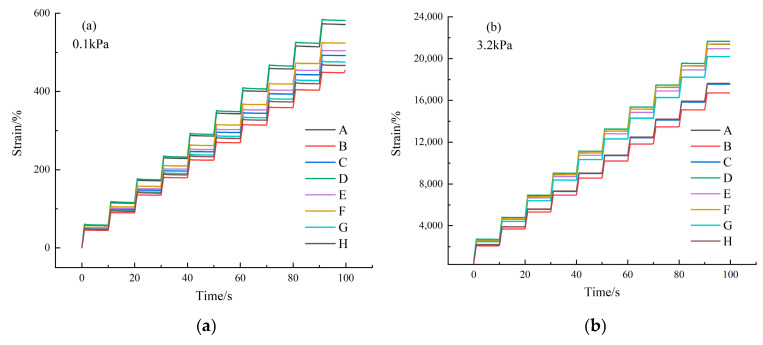
Test results of the MSCR: (**a**) 0.1 kPa and (**b**) 3.2 kPa.

**Figure 8:**
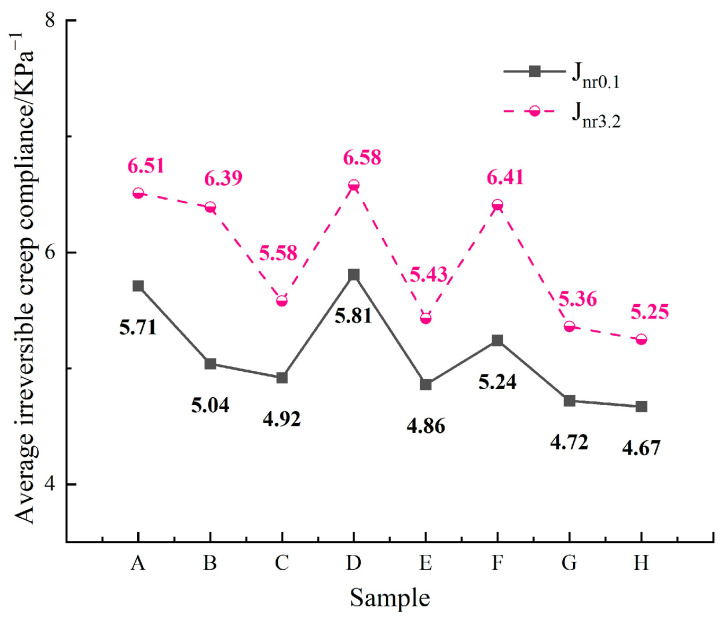
Average irreversible creep compliance.

To more precisely evaluate the high-temperature performance of asphalt binder, an MSCR test was performed. The experimental results are presented in Figure 7, and the average irreversible creep compliance results are shown in Figure 8, where J_nr0.1_ and J_nr3.2_ denote the average irreversible creep compliance at a stress level of 0.1 kPa and 3.2 kPa, respectively.

Generally, lower average irreversible creep compliance indicates better high-temperature performance of the asphalt binder [36]. The MSCR test results indicate that at high temperatures, each asphalt binder sample generates a relatively large strain. The strain increases with the applied stress, while the viscoelastic linearity of the samples progressively decreases. Under the same stress conditions, Sample D generates higher strain compared to the other samples. As shown in Figure 8, the variation trends are consistent across all the asphalt binder samples, regardless of the stress level. The rankings of the J_nr0.1_ and J_nr3.2_ values, from lowest to highest, are as follows: Sample H < Sample G < Sample E. The results of both the DSR and MSCR tests demonstrate that, among the eight asphalt binders, the top three in terms of high-temperature performance are Sample H, Sample G, and Sample E, and the top three in terms of the asphaltene content are Sample H, Sample G, and Sample E. An increase in the asphaltene content results in a higher rutting factor and lower average irreversible creep compliance at elevated temperatures. This is primarily due to the strengthened “skeleton effect” of the polar components in the asphalt binder, which alters its rheological properties under high-temperature conditions.

### 3.4. Low-Temperature Performance of Asphalt Binder

**Figure 9:**
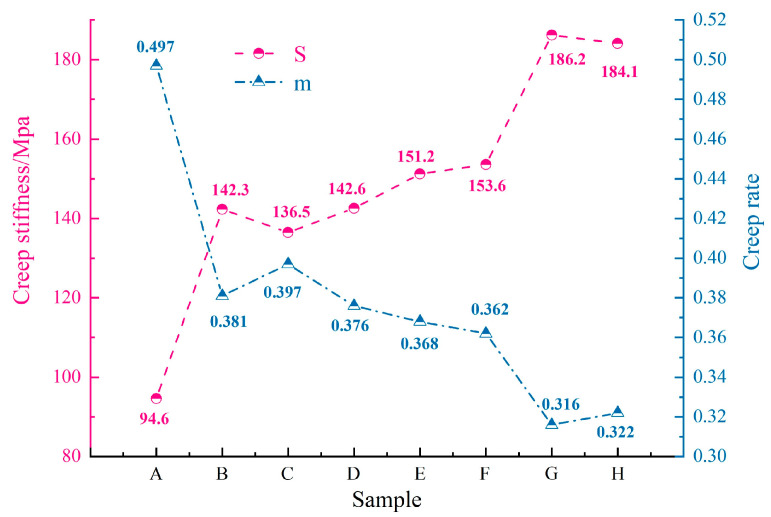
Test results of the BBR.

Most researchers worldwide use the creep stiffness (S) and creep rate (m) as key indicators for assessing the low-temperature performance of asphalt binder [37]. Under the same testing conditions, asphalt binder with better low-temperature performance typically exhibits lower S and higher m values. As presented in Figure 9, the S of the eight asphalt binders is ranked from lowest to highest as follows: Sample A < Sample C < Sample B. The top three asphalt binders in terms of the creep rate (m) are ranked as follows: Sample A > Sample C > Sample B. The analysis of the test results indicates that, among the eight asphalt binders, the top three in terms of low-temperature performance are as follows: Sample A > Sample C > Sample B. The chart analysis reveals that the S negatively correlates with the resin content, positively correlates with the saturate content, negatively correlates with the aromatic content, and positively correlates with the asphaltene content. The correlation trend of the m with the SARA fraction content is opposite to that of the S.

## 4. Discussion

### 4.1. Gray Correlation Analysis

Gray relational analysis (GRA) is an algorithm used to evaluate the degree of correlation between a reference sequence and a main sequence, with no stringent requirements regarding the sample size or data distribution [38]. The steps involved are as follows.

(i) When the measurement units of the influencing factors differ or the value ranges of certain factors vary significantly, the contributions of other factors may be overlooked. Moreover, if the influence directions of these factors on the indicators differ, it may result in inaccurate analysis outcomes. The observation values of each factor must be transformed into comparable sequences, a process known as gray relational generation. The calculation method is presented in Equation (1):

(1) $$f(x_{i}(k))=\frac{x_{i}\left(k\right)}{\bar{x_{i}}}=y_{i}(k)$$

where $x_{i}$ is the reference sequence; $\bar{x_{i}}$ is the mean of the reference sequence; $y_{i}$ is the normalized reference sequence; i = 0, 1, 2, …; and when i = 0, the sequence represents the main sequence. k is an element in a reference sequence, k = 0, 1, 2, ….

(ii) The correlation coefficient γ between each reference sequence and the corresponding elements of the main sequence is calculated using Equations (2)–(5):

(2) $$\gamma (x_{0}\left(k\right),x_{i}(k))=\frac{\Delta min+\rho \Delta max}{\Delta_{ik}+\rho \Delta max}$$

(3) $$\mathrm{where}\;\Delta min=\min(\begin{array}{c} min\\ k \end{array}\left|x_{0}(k)-x_{i}(k)\right|)$$

(4) $$\Delta max=max(\begin{array}{c} max\\ k \end{array}\left|x_{0}(k)-x_{i}(k)\right|)$$

(5) $$\Delta_{ik}=\left|x_{0}(k)-x_{i}(k)\right|$$

where ρ is the distinguishing coefficient, $\rho \in \left[0,1\right]$. The value of ρ was set to 0.5, based on the relevant literature cited in this study [39].

(iii) The calculation of the gray relational coefficient τ is given by Equation (6).

(6) $$\tau (x_{0},x_{i})=\frac{1}{n}\sum_{j=1}^{n}\gamma (x_{0}\;(k),x_{i}\;(k))$$

**Figure 10:**
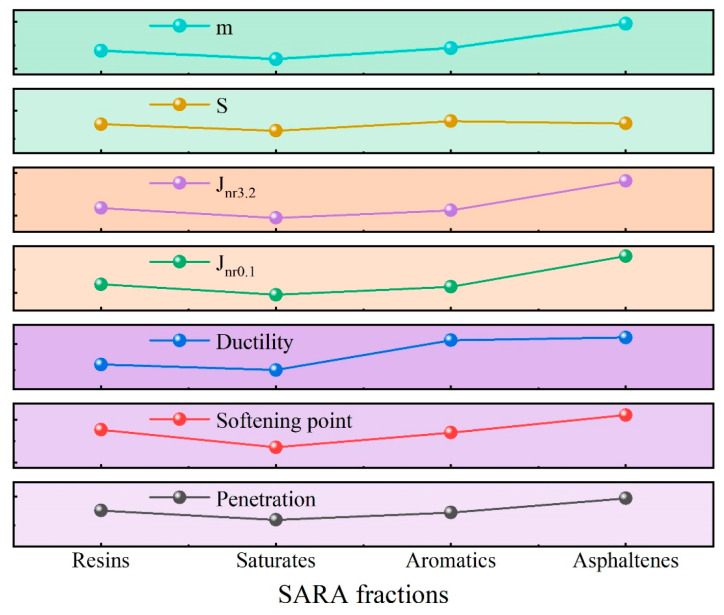
Correlation of the GRA.

The contents of the SARA fractions in the asphalt binder samples were chosen as the reference sequences. The penetration, softening point, ductility, J_nr0.1_, J_nr3.2_, S, and m were used as the main sequence. The calculation results are shown in Table 5 and Figure 10. The evaluation items in the table are arranged in descending order of their degree of association, with higher rankings indicating a stronger correlation with the main sequence. When the high-temperature performance indicators of asphalt binder (penetration, softening point, J_nr3.2_, J_nr0.1_) are considered the main sequence, asphaltene and resin are ranked highly in terms of their correlation. This suggests that the high-temperature performance of asphalt binder is predominantly influenced by its heavier components, and the colloidal structure governed by asphaltene and resin has a direct impact on the material’s high-temperature performance. When the low-temperature performance indicators of asphalt binder (ductility, S, m) are used as the main sequence, the asphaltene and aromatic components exhibit a strong correlation. This indicates that the low-temperature performance of asphalt binder is primarily influenced by the combined effects of both heavy and light components, with asphaltene and aromatic compounds playing a key role.

### 4.2. Pearson Correlation Analysis

The relationship between the contents of the SARA fractions and the asphalt binder performance was examined using Pearson correlation analysis (PCA). The abbreviations for the different indicators are shown in Table 6. In the PCA, R represents the correlation between two variables and is computed using Equation (7). The greater the absolute value of R, the stronger the correlation between the two indicators. The value of R ranges from −1 to 1. Based on the distribution of the $\left|R\right|$ values, the correlations between different variables are categorized into five levels [40], as shown in Table 7.

(7) $$R=\frac{\sum_{i=1}^{n}\left(x_{i}-\bar{x})(y_{i}-\bar{y}\right)}{\sqrt{\sum_{i=1}^{n}{\left(x_{i}-\bar{x}\right)}^{2}}\sqrt{\sum_{i=1}^{n}{\left(y_{i}-y\right)}^{2}}}$$

**Figure 11:**
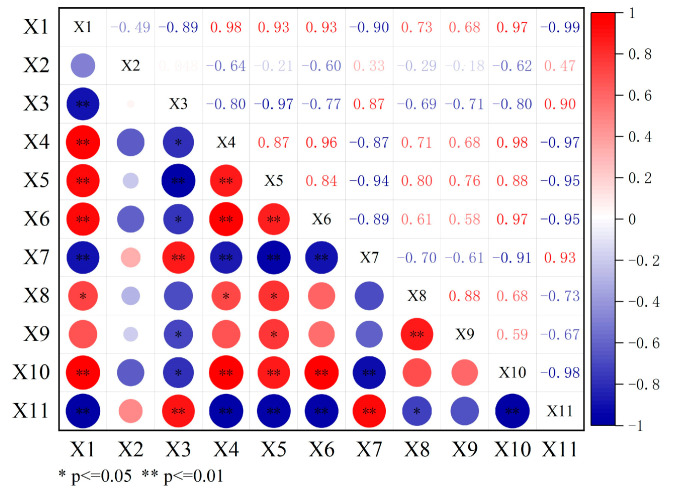
Correlation heat map of the different indicators.

A correlation heat map of the different indicators is shown in Figure 11. The resin content exhibits a positive correlation with the penetration, softening point, J_nr0.1_, J_nr3.2_, and S. The correlation coefficients $\left|R\right|$ for the penetration, softening point, and S are greater than or equal to 0.8, signifying a very high positive correlation, and for the J_nr0.1_ and J_nr3.2_, they range from 0.6 to 0.8, indicating a high positive correlation. The resin content also shows a negative correlation with the ductility and m, for which the $\left|R\right|$ values are greater than or equal to 0.8, indicating a very high negative correlation. The saturate content shows a positive correlation with the ductility and m: the $\left|R\right|$ for the m ranges from 0.4 to 0.6, indicating a moderate positive correlation, and that for the ductility ranges from 0.2 to 0.4, indicating a low positive correlation. The saturate content also shows a negative correlation with the penetration, softening point, J_nr0.1_, J_nr3.2_, and S: The $\left|R\right|$ values for the softening point and S range from 0.6 to 0.8, indicating a high negative correlation. The $\left|R\right|$ values for the penetration and J_nr0.1_ range from 0.2 to 0.4, indicating a low negative correlation. The $\left|R\right|$ for the J_nr3.2_ ranges from 0 to 0.2, indicating a negligible negative correlation. The aromatic content shows a positive correlation with the penetration, ductility, and m, for which the $\left|R\right|$ values are greater than or equal to 0.8, indicating a very high positive correlation. The aromatic content also shows a negative correlation with the softening point, J_nr0.1_, J_nr3.2_, and S. The $\left|R\right|$ values for the softening point and S are greater than or equal to 0.8, indicating a very high negative correlation, and the $\left|R\right|$ values for the J_nr0.1_ and J_nr3.2_ range between 0.6 and 0.8, indicating a high negative correlation. Finally, the asphaltene content shows a positive correlation with the softening point, J_nr0.1_, J_nr3.2_, and S. The $\left|R\right|$ values for the softening point and S are greater than or equal to 0.8, indicating a very high positive correlation, and those for the J_nr0.1_ and J_nr3.2_ range between 0.6 and 0.8, indicating a high positive correlation. The asphaltene content also shows a negative correlation with the penetration, ductility, and S, for which the $\left|R\right|$ values are greater than or equal to 0.8, indicating a very high negative correlation.

Based on the performance testing and correlation analysis, it can be concluded that asphaltene is the component with the highest molecular weight and strongest polarity in asphalt binder. It is typically dispersed in the asphalt binder as colloidal particles, and its primary role is to enhance the structural stability of the asphalt binder. Resin is a component with a relatively high molecular weight and weaker polarity. It stabilizes the asphaltene colloidal particles and acts as a “protective gel layer” for asphaltene. The contents of asphaltene and resin play a decisive role in the high-temperature performance of asphalt binder. Together, they form the colloidal structure of asphalt binder, and through controlling their ratio, the high-temperature performance of the asphalt binder can be optimized. In the expression of asphalt binder’s low-temperature performance, an increase in the heavy components, particularly the asphaltene content, negatively affects the low-temperature properties. However, an increase in the aromatic component content is beneficial for improving low-temperature performance. Therefore, the low-temperature performance of asphalt binder results from the synergistic interaction of both heavy and light components, with aromatic components and asphaltene playing key roles.

## 5. Conclusions

This study used eight virgin asphalt binders, labeled A to H, as raw materials. The contents of the SARA fractions in each asphalt binder were determined using TLC-FID. Additionally, the relevant binder properties of these asphalt binders (including the conventional properties, DSR, and BBR) were measured. Finally, GRA and PCA were employed to investigate the relationship between the component contents and the binder performance. The main findings were as follows.

(1) Both the DSR and MSCR tests indicate that, among the eight asphalt binders, the top three in terms of high-temperature performance are Sample H, Sample G, and Sample E. Additionally, the top three in terms of the asphaltene content are Sample H, Sample G, and Sample E. It can be demonstrated that an increase in the asphaltene content leads to a rise in the rutting factor of asphalt binder under high-temperature conditions, while the J_nr_ decreases. This is primarily due to the enhanced “skeleton effect” formed by the polar components in the asphalt binder, which in turn affects the rheological properties of the asphalt binder material at high temperatures.

(2) When the high-temperature performance indicators of asphalt binder (penetration, softening point, J_nr3.2_, J_nr0.1_) are used as the main sequence, asphaltene and resin are ranked highly in terms of their correlation. This indicates that the high-temperature performance of asphalt binder is primarily determined by its heavy components, with the colloidal structure governed by asphaltene and resin playing a direct role in high-temperature performance. When the low-temperature performance indicators of asphalt binder (ductility, S, m) are used as the main sequence, the asphaltene and aromatic components rank highly in terms of the correlation. This indicates that the low-temperature performance of asphalt binder is the result of the synergistic effects of both heavy and light components, with aromatic components and asphaltene playing key roles.

(3) PCA shows that resin is very highly positively correlated with the penetration (R: 0.93), softening point (R: 0.93), and S (R: 0.97); highly positively correlated with the J_nr0.1_ (R: 0.73) and J_nr3.2_ (R: 0.68); and very highly negatively correlated with the ductility (R: −0.90) and m (R: −0.99). Saturates are moderately positively correlated with the m (R: 0.47); lowly positively correlated with the ductility (R: 0.33); highly negatively correlated with the softening point (R: −0.60) and S (R: −0.62); lowly negatively correlated with the penetration (R: −0.21) and J_nr0.1_ (R: −0.29); and negligibly negatively correlated with the J_nr3.2_ (R: −0.18). Aromatic components are very highly positively correlated with the penetration (R: 0.87), ductility (R: 0.87), and m (R: 0.90); very highly negatively correlated with the softening point (R: −0.77) and S (R: −0.80); and highly negatively correlated with the J_nr0.1_ (R: −0.69) and J_nr3.2_ (R: −0.71). Finally, asphaltene is very highly positively correlated with the softening point (R: 0.96) and S (R: 0.98); highly positively correlated with the J_nr0.1_ (R: 0.71) and J_nr3.2_ (R: 0.68); and very highly negatively correlated with the penetration (R: −0.97), ductility (R: −0.87), and m (R: −0.97).

(4) Based on the performance testing and correlation analysis, it can be concluded that the contents of asphaltene and resin play a decisive role in determining the high-temperature performance of asphalt binder. Together, they form the colloidal structure of asphalt binder, and the high-temperature performance can be optimized by adjusting their ratio. In terms of low-temperature performance, the increase in the heavy components, particularly in the asphaltene content, negatively impacts the low-temperature properties. However, an increase in the aromatic content improves the low-temperature performance.

## Figures and Tables

**Table 1:** Test numbers and basic information on the eight types of virgin asphalt binders.

Test Number	Type	Specification	Manufacturer
A	Petroleum asphalt binder	90 ^#^	SK Group, Seoul, Republic of Korea
B	Petroleum asphalt binder	70 ^#^	SK Group, Seoul, Republic of Korea
C	Petroleum asphalt binder	70 ^#^	Zhejiang Hualian Petrochemical Co., Ltd., Zhejiang, China
D	Petroleum asphalt binder	70 ^#^	China National Offshore Oil Co., Ltd., Beijing, China
E	Petroleum asphalt binder	70 ^#^	Qinhuangdao CPC Petrochemical Co., Ltd., Qinhuangdao, China
F	Petroleum asphalt binder	70 ^#^	Royal Dutch Shell
G	Petroleum asphalt binder	50 ^#^	SK Group, Seoul, Republic of Korea
H	Petroleum asphalt binder	50 ^#^	Zhejiang Hualian Petrochemical Co., Ltd., Beijing, China

“^#^” Indicating the grade of asphalt binder.

**Table 2:** Test reagents.

Reagent Name	Specification	Manufacturer
Heptane	99%	Shanghai Macklin Biochemical Technology Co., Ltd., Shanghai, China
Toluene	98%	Shanghai Macklin Biochemical Technology Co., Ltd., Shanghai, China
Anhydrous ethanol	99.8%	Shanghai Macklin Biochemical Technology Co., Ltd., Shanghai, China

**Table 3:** Test results of the SARA fractions of virgin asphalt binders.

Sample	SARA Fraction Content/%			
	Resins	Saturates	Aromatics	Asphaltenes
A	36.22	21.24	36.29	6.25
B	27.43	36.65	26.41	9.51
C	28.54	37.46	25.62	8.38
D	27.35	38.77	24.6	9.28
E	26.78	35.67	23.77	13.78
F	27.08	33.6	25.12	14.2
G	20.28	31.99	19.61	28.12
H	19.93	33.85	18.72	27.5

**Table 4:** Test results of the DSR.

Temperature/°C	Index	Sample							
		A	B	C	D	E	F	G	H
52	G*/kPa	4.48	7.08	5.78	4.52	7.23	6.84	7.79	8.56
58		2.10	3.07	2.53	1.89	3.31	2.95	3.72	4.08
64		0.94	1.40	1.14	0.91	1.61	1.54	1.77	1.97
70		0.49	0.68	0.57	0.48	0.84	0.82	0.88	0.95
76		0.25	0.35	0.32	0.25	0.45	0.43	0.48	0.51
82		0.14	0.19	0.17	0.15	0.23	0.23	0.24	0.26
52	δ/°	82.4	82.8	85.5	85.5	76.9	74.8	78.2	79.6
58		84.4	84.7	86.9	86.4	78.6	76.5	80.3	82.0
64		85.6	86.2	87.6	87.1	80.5	79.1	82.2	84.2
70		87.1	87.5	87.9	87.8	82.8	82.2	84.2	86.2
76		86.7	88.7	88.8	87.4	85.2	85.0	86.1	87.9
82		88.0	89.7	88.7	89.0	87.4	87.2	87.7	89.5

**Table 5:** Calculation results of the GRA.

Main Sequence	Reference Sequence	Correlation	Ranking
Penetration	Resins	0.702	2
	Saturates	0.637	4
	Aromatics	0.688	3
	Asphaltenes	0.787	1
Softening point	Resins	0.706	2
	Saturates	0.542	4
	Aromatics	0.679	3
	Asphaltenes	0.843	1
Ductility	Resins	0.642	3
	Saturates	0.599	4
	Aromatics	0.83	2
	Asphaltenes	0.852	1
J_nr0.1_	Resins	0.475	2
	Saturates	0.385	4
	Aromatics	0.453	3
	Asphaltenes	0.715	1
J_nr3.2_	Resins	0.472	2
	Saturates	0.38	3
	Aromatics	0.45	4
	Asphaltenes	0.724	1
S	Resins	0.703	3
	Saturates	0.657	4
	Aromatics	0.725	1
	Asphaltenes	0.708	2
m	Resins	0.554	3
	Saturates	0.481	4
	Aromatics	0.576	2
	Asphaltenes	0.784	1

**Table 6:** The abbreviations for the different indicators.

Indicator	Abbreviation	Indicator	Abbreviation
Resins	X1	Ductility	X7
Saturates	X2	J_nr0.1_	X8
Aromatics	X3	J_nr3.2_	X9
Asphaltenes	X4	S	X10
Penetration	X5	m	X11
Softening point	X6		

**Table 7:** Pearson’s correlation coefficients (R) and their degree and interpretation.

$\left|R\right|$	Interpretation
$\left|R\right|$ ≥ 0.8	Very high correlation
0.6 ≤ $\left|R\right|$ < 0.8	High correlation
0.4 ≤ $\left|R\right|$ < 0.6	Moderate correlation
0.2 ≤ $\left|R\right|$ < 0.4	Low correlation
0 ≤ $\left|R\right|$ < 0.2	Negligible correlation

## Data Availability

Data are contained within the article.
